# Donor age and *C1orf132/MIR29B2C* determine age-related methylation signature of blood after allogeneic hematopoietic stem cell transplantation

**DOI:** 10.1186/s13148-016-0257-7

**Published:** 2016-09-06

**Authors:** Magdalena Spólnicka, Renata Zbieć Piekarska, Emilia Jaskuła, Grzegorz W. Basak, Renata Jacewicz, Agnieszka Pięta, Żanetta Makowska, Maciej Jedrzejczyk, Agnieszka Wierzbowska, Agnieszka Pluta, Tadeusz Robak, Jarosław Berent, Wojciech Branicki, Wiesław Jędrzejczak, Andrzej Lange, Rafał Płoski

**Affiliations:** 1 Biology Department, Central Forensic Laboratory of the Police, Warsaw, 00-583 Poland; 2 L. Hirszfeld Institute of Immunology and Experimental Therapy, Polish Academy of Sciences, Wroclaw, 53-114 Poland; 3 Lower Silesian Center for Cellular Transplantation with National Bone Marrow Donor Registry, Wroclaw, 53-439 Poland; 4 Department of Hematology, Oncology and Internal Diseases, The Medical University of Warsaw, Warsaw, 02-097 Poland; 5 Department of Forensic Medicine, Medical and Forensic Genetics Laboratory, Medical University of Lodz, Lodz, 91-304 Poland; 6 Department of Hematology, Medical University of Lodz, Copernicus Memorial Hospital, Lodz, 93-510 Poland; 7 Malopolska Centre of Biotechnology, Jagiellonian University, Krakow, 30-387 Poland; 8 Department of Medical Genetics, Warsaw Medical University, Pawińskiego 3c, Warsaw, PL 02-106 Poland

**Keywords:** Allogeneic hematopoietic stem cell transplantation, DNA methylation, Aging, Rejuvenation, MIR29B2C

## Abstract

**Background:**

Our recent study demonstrated that DNA methylation status in a set of CpGs located in *ELOVL2*, *C1orf132*, *TRIM59*, *KLF14*, and *FHL2* can accurately predict calendar age in blood. In the present work, we used these markers to evaluate the effect of allogeneic hematopoietic stem cell transplantation (HSCT) on the age-related methylation signature of human blood.

**Methods:**

DNA methylation in 32 CpGs was investigated in 16 donor-recipient pairs using pyrosequencing. DNA was isolated from the whole blood collected from recipients 27–360 days (mean 126) after HSCT and from the donors shortly before the HSCT.

**Results:**

It was found that in the recipients, the predicted age did not correlate with their calendar age but was correlated with the calendar age (*r* = 0.94, *p* = 4 × 10^−8^) and predicted age (*r* = 0.97, *p* = 5 × 10^−10^) of a respective donor. Despite this strong correlation, the predicted age of a recipient was consistently lower than the predicted age of a donor by 3.7 years (*p* = 7.8 × 10^−4^). This shift was caused by hypermethylation of the *C1orf132* CpGs, for *C1orf132* CpG_1. Intriguingly, the recipient-donor methylation difference correlated with calendar age of the donor (*r* = 0.76, *p* = 6 × 10^−4^). This finding could not trivially be explained by shifts of the major cellular factions of blood.

**Conclusions:**

We confirm the single previous report that after HSCT, the age of the donor is the major determinant of age-specific methylation signature in recipient’s blood. A novel finding is the unique methylation dynamics of *C1orf132* which encodes MIR29B2C implicated in the self-renewing of hematopoietic stem cells. This observation suggests that *C1orf132* could influence graft function after HSCT.

**Electronic supplementary material:**

The online version of this article (doi:10.1186/s13148-016-0257-7) contains supplementary material, which is available to authorized users.

## Introduction

It has been shown in animal models that elderly individuals exposed to a young systemic environment, for example, by surgical connection of the circulatory systems of young and old animal (heterochronic parabiosis), show reduced signs of biological aging in the cardiovascular, skeletal, and gastrointestinal as well as central nervous system [1–3]. Whereas parabiosis is a strictly experimental system, it has certain similarities with clinical state during an allogeneic transplantation including bone marrow transplant or the more often performed peripheral blood hematopoietic stem cell transplantation (HSCT).

Recently, we described a set of five CpG sites whose methylation in the whole blood predicts calendar age with high accuracy [4]. This set of predictors included CpG sites in *ELOVL2* (6p24.2), *C1orf132* (1q32.2), *TRIM59* (3q25.33), *KLF14* (7q32.3), and *FHL2* (2q12.2). We developed a convenient method based on pyrosequencing for robust DNA methylation analysis in 32 CpGs located in these loci including the five best CpG sites included in the prediction model. Our approach allowed to predict actual age with mean absolute deviation (MAD) of 3.9 years and number of correct predictions (+/−5 years) that ranged depending on age category, from 87 to 50 % [4].

The aim of this study was to evaluate the effect of HSCT on the age-related methylation signature of human blood obtained with set of 5 CpG markers of age included in our age prediction model. In particular, we examined the effects of donor and recipient age as well as assessed whether differences existed in post-transplant methylation dynamics among individual CpG markers.

## Methods

### Materials

We studied peripheral blood samples from 16 pairs of donors and recipients. Transplant indications were in line with the European Group for Blood and Marrow Transplantation (EBMT) guidelines and the searching process was conducted according to the World Marrow Donor Association (WMDA) recommendations. Eleven patients suffered from acute myeloblastic leukemia, two from myeloproliferative diseases, one from T-cell acute lymphoblastic leukemia, one from chronic myeloblastic leukemia, and one from paroxysmal nocturnal hemoglobinuria. Fourteen pairs were of the same sex; two males received a transplant from female donors. Samples from the donors included original samples collected for the purpose of HLA typing within 6 months before alloHSCT. Samples from the recipients reconstituted with hematopoietic stem cells of donor origin were collected at the median time of 91 days (range, 27–360) after alloHSCT. The general characteristics of recipients and donors are shown in Table 1. Briefly, the median age of recipients at the time of alloHSCT was 45 (range, 18–59, a detailed list of donors’ and matched recipients’ age is given in Additional file 1: Table S1). Twelve patients received myeloablative and four reduced-intensity conditioning. All patients underwent transplantation from unrelated donors. All patients but one were matched with recipients in 10/10 HLA alleles. All patients received peripheral blood stem cells as a transplant material. The median age of stem cell donors was 28 years (range, 20–63). The graft-versus-host disease prophylaxis included standard doses of cyclosporine (all patients) and methotrexate (12 patients) or mycophenolate mofetil (4 patients). Engraftment was achieved by day +20 in all the patients. In all recipients, full chimerism at the time of blood sample collection for methylation status analysis was demonstrated by tests performed at respective clinical centers. In addition, in all DNA samples, the 100 % chimerism was confirmed by analyzing genotypes of 27 hypervariable STRs using Fusion 6C kit (*Promega*).

**Table 1:** Characteristics of HSCT recipients and donors

	Mean	Standard deviation	Median	Min.	Max.	Lower quartile	Upper quartile
Recipients (*N* = 16. 37.5 % of females)							
Calendar age	42.0	11.5	45	18	59	36	50
Donors (*N* = 16. 50 % of females)							
Calendar age	33.9	13.3	28	20	63	25	40
Period (days) between HSCT and blood sampling in the recipient	125.9	97.5	91	27	360	41	202
Calendar age difference (recipient-donor)	8.1	16.8	11	−19	28	−9	22

### DNA methylation analysis

DNA methylation analysis was performed as described previously [4]. Briefly, 2 ug of sample DNA was bisulfite converted using the Qiagen 96-well kit (Qiagen, Hilden, Germany). PCR reactions were carried out in a total volume of 25 mL to amplify 5 loci in a total volume of 25 mL (6p24.2), *C1orf132* (1q32.2), *TRIM59* (3q25.33), *KLF14* (7q32.3), and *FHL2* (2q12.2). Negative PCR controls were included in each PCR amplification. Pyrosequencing was performed using Pyro Gold reagents on a PyroMark vacuum prep workstation and a PyroMark Q24 instrument, following the manufacturer’s instructions. The generated pyrograms were automatically analyzed using PyroMark analysis software (Qiagen, Hilden, Germany). Our approach allowed to define the methylation status of 32 CpG (7 CpGs in *ELOVL2*, 10 CpG in *FHL2*, 8 CpG in *TRIM59*, 4 CpG in *KLF14*, and 3 CpGs in *C1orf132*). The positions of these CpG are given in Table 1 in [4].

### Statistical analysis

Correlations were assessed by calculating the Pearson correlation coefficients (*r*). We also show *r*^2^ values which indicate the fraction of variance of one variable explained by the other variable as well as *p* values. The differences in the predicted age and in the methylation status in the donor-recipients pairs were analyzed with paired *t* test. In the comparison of methylation status of individual CpG between donors and recipients, Bonferroni correction was applied with the correction factor of 32 (i.e., number of all analyzed CpG). Multivariate analysis was performed with multiple linear regression (lymphocyte numbers and the increase in *C1orf132* CpG_1 methylation were independent variables; donor’s age was the dependent variable). All calculations were performed with *Statistica* software package (StatSoft, Tulsa, OK, USA).

## Results

### Accuracy of age prediction among donors

**Fig. 1:**
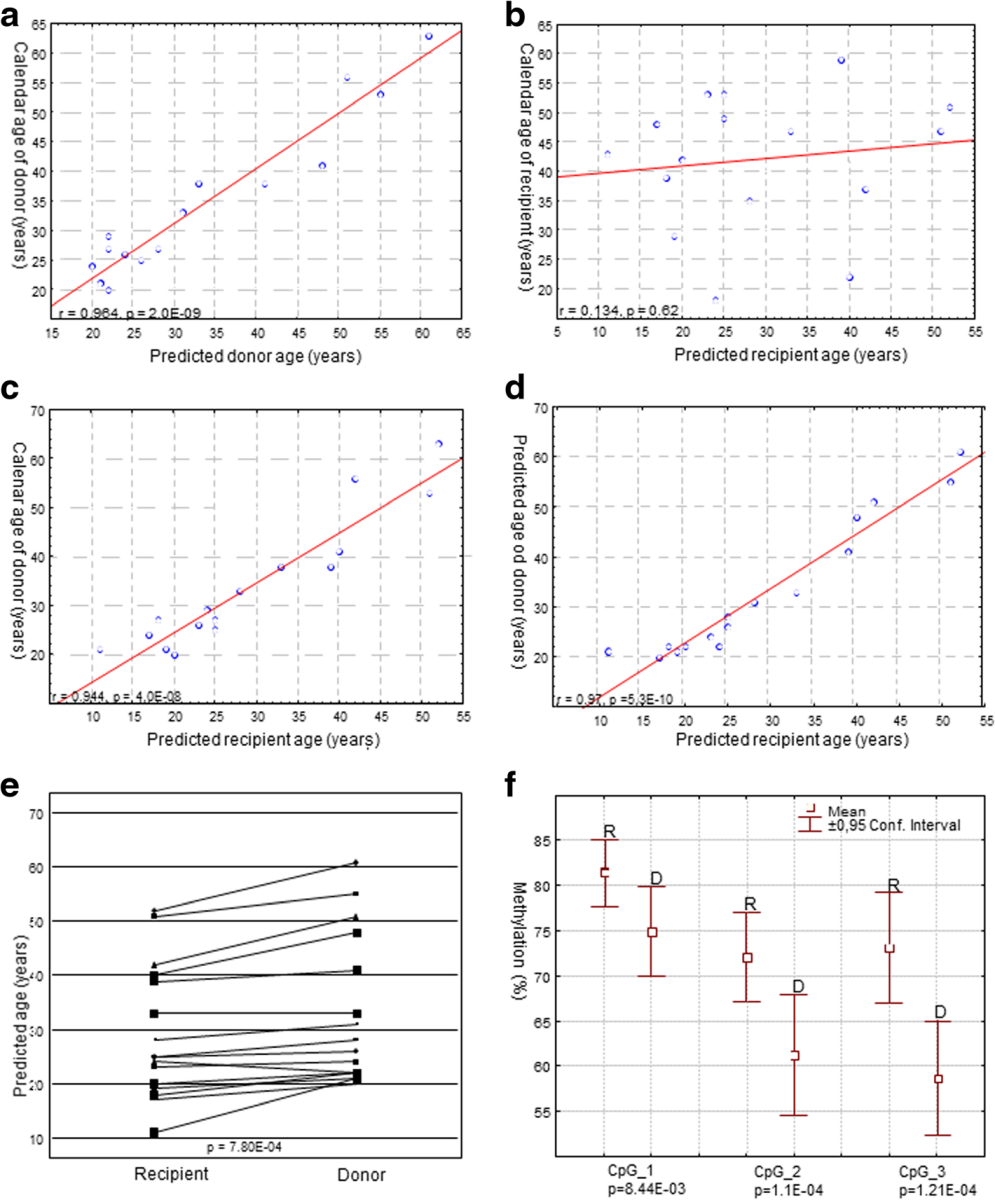
Correlation between age predicted from methylation and calendar age in HSCT donors (**a**) and recipients (**b**). *r* Pearson correlation coefficient, *p* associated *p* value. Correlation between the predicted age of HSCT recipient and calendar age of HSCT donor (**c**) and predicted age of the donor (**d**). Age predicted from methylation in the HSCT recipient-donor pairs; *p* value was calculated by *t* test for paired samples (**e**). Methylation levels of the studied *C1orf132* CpGs in HSCT recipients (R) and donors (D); *p* values were calculated with paired *t* test with Bonferroni correction (**f**)

Among donors, there was a strong correlation between the calendar age and the predicted age (*r* = 0.96, *r*^2^ = 0.93, *p* = 2 × 10^−9^, Fig. 1a). Mean absolute deviation (MAD) was 3 years with min. and max. of 0 and 7 and quartile range from 1.5 to 5 years. The calendar age of 14 out of 16 donors (87.5 %) was predicted with an absolute error of 5 years or less. The mean difference between calendar and predicted age was 1 year, with min. and max. of −7 and 7 years, and quartile range from −1.5 to 4.5 years.

Among HSCT recipients, the predicted age did not depend on the recipient calendar age but strongly correlated with calendar and predicted age of the donor.

Among recipients post HSCT, there was no correlation between the calendar and the age predicted from the methylation analysis of the blood DNA (*r* = 0.134, *r*^2^ = 0.018, *p* = 0.62, Fig. 1b). Of note, analysis of the recipients’ blood revealed that there was a strong correlation between the predicted age assessed by the methylation signature of blood DNA with the calendar age of the donor (*r* = 0.94, *r*^2^ = 0.89, *p* = 4 × 10^−8^, Fig. 1c) as well as the predicted age of the donor assessed by analysis of the donor’s blood (*r* = 0.97, *r*^2^ = 0.94, *p* = 5 × 10^−10^, Fig. 1d).

### The methylation level indicates lower predicted age in the recipients compared to the predicted age in donors after HSCT

Despite the very strong correlation between predicted age of the recipient and the predicted age of the donor, we noted that in all but one case the age predicted from the post-HSCT methylation signature of recipient’s blood was lower than the age predicted from the methylation signature in the donor of transplanted material (Fig. 1e). In the case which constituted the exception the age of the recipient predicted from post HSCT DNA methylation status was 2 years higher than the age predicted from DNA methylation status in the respective donor.

The mean predicted age among recipients was 29.2 years with standard deviation (SD) of 12.3; whereas among the donors, the respective value was 32.9 years with SD of 13.81. The mean difference between the predicted age of the donor and the predicted age of the recipient was 3.7 years with SD of 3.52 (*p* = 0.00078, *t* test for paired samples).

The apparent lower predicted age in the recipients compared to the predicted age in donors seen at the DNA methylation level (i.e., the difference between the predicted age of donor and the predicted age of recipient) did not depend on sex (*p* = 0.49), the age of recipients (*p* = 0.95), the length of period between HSCT and blood sampling in the recipients group (*p* = 0.82), or the difference in the calendar age between the donors and the recipients (*p* = 0.14). However, we noted a trend (*p* = 0.064) towards a correlation of the size of difference between predicted age in the recipients and donors, with donor’s age (*r* = 0.47) suggesting that this effect may be more pronounced when HSCT comes from an older donor (i.e., the older the donor the greater the difference between the his/her predicted age and the predicted age of the recipient).

### The apparent lower predicted age in the recipients compared to the predicted age in donors after HSCT is driven by selective hypermethylation of CpGs within the *C1orf132* locus

We further investigated whether the consistent difference of ~4 years between the predicted age of recipient and the predicted age of donor was caused by coherent effects of all CpG predictors or the direction of DNA methylation changes was different in particular loci. In Additional file 1: Table S2, we show the results of comparison of recipient vs. donor methylation differences for all the 5 CpG included in the age prediction model as well as for the remaining 27 CpGs whose methylation was assessed in our study. We found that among the CpG sites included in the model, only CpG_1 at *C1orf132* (*C1orf132* CpG_1) showed different methylation among recipients vs. donors (mean difference 8.2 %; SD = 6.9 % (*p* = 0.00026; *P*_corrected_ = 0.008).

In order to better estimate the size of this effect, we developed an age prediction model based solely on CpGs in *C1orf132*. The model included *C1orf132* CpG_1 and *C1orf132* CpG_3, and it had a reasonable performance (*r* = 0.89, *r*^2^ = 0.8, and MAD = 6.1 years). Using the “*C1orf132* only” model, the mean predicted age of recipients was 22.4 years and that of donors was 33.8 years, yielding a difference of 11.3 years (*p* = 0.000016, paired *t* test).

Although we did not detect individual recipient-donor DNA methylation differences in other loci than *C1orf132*, these loci could still have weak effects possibly amounting to detectable joint influence. In order to test this, we developed an age prediction model including all loci except the *C1orf132* (*r* = 0.96, *r*^2^ = 0.92, MAD = 4.1 years). Using this “non-*C1orf132* model” the mean predicted age of recipients was 32.1 years and that of donors was 33.1 years, yielding a difference of 1.1 year which was not statistically significant (*p* = 0.28, paired *t* test).

### Size of the increase in methylation of *C1orf132* CpG_1 in the recipient vs. donor correlates with calendar age of the donor

The size of the increase in methylation of *C1orf132* CpG_1 in the recipients vs. the donors correlated with the calendar age of the donors (*r* = 0.76, *r*^2^ = 0.58, *p* = 0.0006) but not with sex (*r* = −0.40, *p* = 0.12), the recipient’s age (*r* = 0.13, *p* = 0.62), the length of time between HSCT and blood sampling in the recipient group (*r* = −0.004, *p* = 0.99), or diagnosis (AML vs. other, 9 vs. 6.4 years, respectively, *p* > 0.5). The size of the increase in methylation of *C1orf132* CpG_1 in the recipients vs. the donors nominally correlated also with the recipient-donor calendar age differences (*r* = −0.51, *p* = 0.043), but this effect was no longer observed after adjustment for calendar age of the donors (*p* = 0.7). Correlation with the age of the donors was also found with the recipient vs. donor age differences calculated using the “*C1orf132* only” model (*r* = −0.7, *r*^2^ = 0.5, *p* = 0.003).

The increase in the methylation in the recipients vs. the donors was also apparent for two other *C1orf132* CpGs studied; for *C1orf132* CpG_2, the difference was 13.4 % (SD = 7.54. *p* = 0.000003, *P*_corrected_ = 0.0001) and for *C1orf132* CpG_3 it was 13.8 % (SD = 7.8. *p* = 0.000004, *P*_corrected_ = 0.00012, Fig. 1f). However, no statistically significant correlation was observed between the size of these effects and the donor’s age or other analyzed variables (data not shown).

The apparent effect of the donor’s age on the methylation of *C1orf132* CpG_1 in the recipient group could be secondary to the differences in the cellular composition of the reconstituted hematopoietic system in the recipients receiving transplant from young vs. older donors. Indeed, when we analyzed donors age vs. white blood cell (WBC) or counts/percentages of granulocytes/lymphocytes/monocytes in the recipients, we found that donor’s age correlated with lymphocyte numbers in the recipient group (*r* = 0.78, *r*^2^ = 0.61, *p* = 0.0003). Since lymphocyte numbers in the recipient also correlated with the size of the increased *C1orf132* CpG_1 methylation in the recipient vs. donor (*r* = 0.63, *r*^2^ = 0.40, *p* = 0.009), we analyzed the effect of lymphocyte numbers and the increase in *C1orf132* CpG_1 methylation on donor’s age by multiple linear regression. We found that lymphocyte numbers and the difference in *C1orf132* CpG_1 methylation between the recipient and donor were independent predictors of donor’s age (beta = 0.51, *p* = 0.016 and beta = 0.44, *p* = 0.033, respectively).

## Discussion

Among human HSCT donors and recipients, we studied methylation in 5 CpGs recently validated by our group for age estimation. We found that the methylation signature of blood post HSCT does not depend on the recipient age but is strongly correlated with the calendar age of the donor. However, despite this correlation we observed that the age of HSCT recipient as predicted from DNA methylation was lower than the age predicted in donors on average by 4 years. Detailed analysis of methylation status of all studied CpG (*N* = 32) showed that this discrepancy was caused by apparently individual effects of CpGs at the *C1orf132* locus.

The age prediction among HSCT donors (who can be regarded as healthy subjects representative of general population) independently confirms good performance of our model [4]. The quality of age prediction achieved in the present study (MAD = 3 years, 87.5 % correct predictions) was comparable to that originally reported (MAD = 3.9, 71.7 % correct predictions) [4], especially when it was taken into account that majority of the donors were relatively young belonging to age category 20–39 years for which the performance of our model was better (MAD 3.3, 76.7 % correct predictions) than for older age groups [4].

The observation that the age of HSCT recipient predicted by post-transplant DNA methylation analysis performed on DNA isolated from whole blood correlates with donor’s but not recipient’s age confirms the results of the recent study by Weidner et al. [5]. Both studies are consistent in the conclusion that the hematopoietic niche of the recipient does not noticeably affect age-associated DNA methylation in blood. It should be emphasized that none of the five CpGs used in our study overlap with any of the three CpG sites analyzed by Weidner et al. [5] which argues that the observed effect is likely to be robust to the exact identity of the set CpG markers used for age prediction.

Despite the very strong correlation between the age predicted for blood in the donor and the recipient, we found that the latter was consistently lower than the former with an exception observed only in a single pair. The difference was relatively small (mean 3.7 years) but it was highly statistically significant. The lower predicted age in the recipients compared to the predicted age in donors by ~4 years is directly opposite to the observation of Weidner et al. [5] who reported that the epigenetic age predictions were on average 7 years *higher* than the calendar age of the donor and attributed this finding to stimulation of hematopoietic stem cells in the donor. One difference between the two studies is the time period between HSCT and the recipient’s blood sampling: in the study of Weidner et al. [5], it was 1 year whereas in our study it varied from 1 month to 1 year with a mean of 3 months. However, we think it is unlikely that this difference accounts for the observed discrepancy because we did not observe any correlation between the length of time between HSCT and blood sampling in the recipients and the size of the difference between predicted age in the recipients and the donors. Such a correlation should be apparent if the aging of the transplanted hematopoietic cells in the donor was indeed fast enough to shift methylation signature up by 7 years over the time of 12 months as observed by Weidner et al. [5].

Whereas the reasons for the discrepancy between our results and those of Weidner et al. [5] are not clear, it is possible that CpG marker selection is important. When we analyzed CpGs individually we found that the lower predicted age in the recipients compared to the donors after HSCT was exclusively caused by hypermethylation of a single CpG (CpG_1 from the *C1orf132* locus) in the recipient vs. donor whereas the remaining four CpG from other loci used for age prediction did not show changes. Furthermore, the remaining two CpGs from the *C1orf132* locus (which were typed but not used for age prediction) showed even stronger hypermethylation suggesting that the effect was present among all *C1orf132* CpGs. This together with the absence of a similar effect among the total of the 29 CpG from the remaining four loci argues against technical artifacts and indicates that genuine differences exist in post-HSCT behavior among loci regarding age-dependent methylation. Whereas potentially interesting from a biological perspective, the dissociation of *C1orf132* methylation from calendar age in certain circumstances may decrease the practical usefulness of this locus for age prediction.

Intriguingly, the size of hypermethylation of *C1orf132* CpG_1 in the recipients was directly correlated with the calendar age of the donors—the older the donor, the higher the methylation. Since *C1orf132* methylation *decreases* with calendar age, the increased methylation could suggest that at this particular CpG HSCT induces “rejuvenation” which gets stronger with the increasing age of the donor. However, since HSCT from older donors is less successful than from younger donors [6], it is unlikely that what we observe is a genuine rejuvenation with positive clinical consequences. Rather than that we speculate that *C1orf132* hypermethylation in recipients of HSCT from older donors reflects some problems with reconstitution of hematopoietic system to the state in which it was present in the donor. This may be linked with delayed or failed restoration of certain types of cells or certain states of their chromatin. It is also possible that senescence-associated methylation changes contribute to the observed effect.

The *C1orf132* has recently been shown to encode a long no-coding RNA (lncRNA) which at its 3′ end includes microRNA MIR29B2C [7]. Whereas the *C1orf132* function remains unclear, it is intriguing that in mice, the miR29 family (miR29ab1 and miR29b2c) has been implicated in the self-renewing ability of HSCs as well as determination of organ and body size [8]. Our results suggest that the *C1orf132/MIR29B2C* locus could also be important for graft function after HSCT and thus should be studied further as a potential graft function/prognostic marker.

Due to relatively small number of studied subjects our study had limitations. Whereas in our data the correlations between donor’s age on one side and *C1orf132* methylation and lymphocyte numbers on the other side appeared independent from each other definite conclusions are difficult. Furthermore, we cannot exclude that *C1orf132* methylation is a marker of a subset of cells whose development primarily depends on the donor’s age and which were not specifically analyzed by us. Studies of these issues are warranted in the future. Finally, our study may not have had sufficient power to detect a significant correlation between the differences in predicted age of donors vs. recipients and the time length from HSCT to blood sample collection.

In conclusion, using an independent set of CpG markers, we confirm that after HSCT the age of the donor is the major determinant of age-specific methylation signature in recipient’s blood whereas the age of recipient does not exert a detectable effect. As a novel finding, we show that in HSCT setting, methylation at *C1orf132* has unique dynamics and depends on the age of the donor. The methylation at *C1orf132* locus should be interesting for further studies of HSCT prognostic markers.

## Conclusions

After HSCT, the age of the donor is the major determinant of age-specific methylation signature in recipient’s blood.

After HSCT methylation of *C1orf132*, which encodes MIR29B2C implicated in the self-renewing of hematopoietic stem cells, it has a unique dynamics suggesting that this locus could influence graft function.

## Additional file

Additional file 1: Table S1. Calendar age of recipients and matched donors. **Table S2**. Methylation (%) of all studied CpGs in recipients and donors. (DOCX 21 kb)

## Abbreviations

BM: bone marrow
HSCT: hematopoietic stem cell transplantation
lncRNA: long no-coding RNA
