# Erratum to: Donor age and *C1orf132/MIR29B2C* determine age-related methylation signature of blood after allogeneic hematopoietic stem cell transplantation

**DOI:** 10.1186/s13148-016-0289-z

**Published:** 2016-11-18

**Authors:** Magdalena Spólnicka, Renata Zbieć Piekarska, Emilia Jaskuła, Grzegorz W. Basak, Renata Jacewicz, Agnieszka Pięta, Żanetta Makowska, Maciej Jedrzejczyk, Agnieszka Wierzbowska, Agnieszka Pluta, Tadeusz Robak, Jarosław Berent, Wojciech Branicki, Wiesław Jędrzejczak, Andrzej Lange, Rafał Płoski

**Affiliations:** 1 Biology Department, Central Forensic Laboratory of the Police, Warsaw, 00-583 Poland; 2 L. Hirszfeld Institute of Immunology and Experimental Therapy, Polish Academy of Sciences, Wroclaw, 53-114 Poland; 3 Lower Silesian Center for Cellular Transplantation with National Bone Marrow Donor Registry, Wroclaw, 53-439 Poland; 4 Department of Hematology, Oncology and Internal Diseases, The Medical University of Warsaw, Warsaw, 02-097 Poland; 5 Department of Forensic Medicine, Medical and Forensic Genetics Laboratory, Medical University of Lodz, Lodz, 91-304 Poland; 6 Department of Hematology, Medical University of Lodz, Copernicus Memorial Hospital, Lodz, 93-510 Poland; 7 Malopolska Centre of Biotechnology, Jagiellonian University, Krakow, 30-387 Poland; 8 Department of Medical Genetics, Warsaw Medical University, Pawińskiego 3c, Warsaw, PL 02-106 Poland

## Erratum

Following publication of this article [1], it has come to our attention that the affiliations for author Andrzej Lange were captured incorrectly, as being affiliated with only ^3^Lower Silesian Center for Cellular Transplantation with National Bone Marrow Donor Registry, Wroclaw 53–439, Poland. The correct affiliation is ^2^L. Hirszfeld Institute of Immunology and Experimental Therapy, Polish Academy of Sciences, Wroclaw 53–114, Poland and ^3^Lower Silesian Center for Cellular Transplantation with National Bone Marrow Donor Registry, Wroclaw 53–439, Poland. The correct affiliations for each author can be seen above in the author list.
