# Supplementary material for: Donor age and C1orf132/MIR29B2C determine age-related methylation signature of blood after allogeneic hematopoietic stem cell transplantation
Source: Clin Epigenetics. 2016 Sep 6;8(1):93. doi: 10.1186/s13148-016-0257-7 (PMC5012039; doi:10.1186/s13148-016-0257-7)
Supplement: Additional file 1: Table S1. — Calendar age of recipients and matched donors. Table S2. Methylation (%) of all studied CpGs in recipients and donors. (DOCX 21 kb) [file 13148_2016_257_MOESM1_ESM.docx]

| **Supplementary tables**  **Supplementary table 1.** Calendar age of recipients and matched donors   \|  \| Age \|  \| \| --- \| --- \| --- \| \| id \| Recipient \| Donor \| \| 2 \| 47 \| 53 \| \| 4 \| 22 \| 41 \| \| 6 \| 53 \| 25 \| \| 9 \| 35 \| 33 \| \| 13 \| 47 \| 38 \| \| 17 \| 51 \| 63 \| \| 20 \| 29 \| 21 \| \| 22 \| 37 \| 56 \| \| 26 \| 18 \| 29 \| \| 28 \| 48 \| 24 \| \| 30 \| 39 \| 27 \| \| 34 \| 53 \| 26 \| \| 36 \| 49 \| 27 \| \| 42 \| 42 \| 20 \| \| 44 \| 43 \| 21 \| \| 48 \| 59 \| 38 \|   **Supplementary Table 2.** Methylation (%) of all studied CpGs in recipients and donors | | | | | | | |
| --- | --- | --- | --- | --- | --- | --- | --- | --- | --- | --- | --- | --- | --- | --- | --- | --- | --- | --- | --- | --- | --- | --- | --- | --- | --- | --- | --- | --- | --- | --- | --- | --- | --- | --- | --- | --- | --- | --- | --- | --- | --- | --- | --- | --- | --- | --- | --- | --- | --- | --- | --- | --- | --- | --- | --- | --- | --- | --- | --- | --- | --- |
|  |  |  |  |  |  |  |  |
| CpG |  | Mean | Std.Dv. | Difference | Std.Dv. | p | p corr. |
|  |  |  |  | (rec-don.) | Diff. |  | (N=32) |
| ELOVL2_C1 | recipient | 33,81 | 8,60 |  |  |  |  |
|  | donor | 33,81 | 7,68 | 0,00 | 5,38 | 1,000000 |  |
|  |  |  |  |  |  |  |  |
| ELOVL2_C2 | recipient | 28,00 | 7,55 |  |  |  |  |
|  | donor | 29,94 | 7,76 | -1,94 | 6,12 | 0,224359 | NS |
|  |  |  |  |  |  |  |  |
| ELOVL2_C3 | recipient | 59,69 | 8,28 |  |  |  |  |
|  | donor | 61,56 | 8,71 | -1,88 | 3,65 | 0,057700 | NS |
|  |  |  |  |  |  |  |  |
| ELOVL2_C4 | recipient | 40,31 | 10,27 |  |  |  |  |
|  | donor | 40,81 | 9,97 | -0,50 | 3,81 | 0,607512 | NS |
|  |  |  |  |  |  |  |  |
| ELOVL2_C5 | recipient | 27,63 | 7,93 |  |  |  |  |
|  | donor | 27,81 | 7,48 | -0,19 | 4,92 | 0,880765 | NS |
|  |  |  |  |  |  |  |  |
| ELOVL2_C6 | recipient | 55,06 | 10,45 |  |  |  |  |
|  | donor | 55,13 | 10,31 | -0,06 | 4,99 | 0,960674 | NS |
|  |  |  |  |  |  |  |  |
| **ELOVL2_C7** | recipient | **65,75** | **10,34** |  |  |  |  |
|  | donor | **67,69** | **9,90** | **-1,94** | **3,73** | **0,055446** | NS |
|  |  |  |  |  |  |  |  |
| FHL2_C1 | recipient | 36,63 | 6,67 |  |  |  |  |
|  | donor | 35,63 | 6,67 | 1,00 | 3,35 | 0,250553 | NS |
|  |  |  |  |  |  |  |  |
| **FHL2_C2** | recipient | **40,25** | **6,16** |  |  |  |  |
|  | donor | **39,63** | **6,40** | **0,63** | **3,03** | **0,422313** | NS |
|  |  |  |  |  |  |  |  |
| FHL2_C3 | recipient | 36,38 | 5,46 |  |  |  |  |
|  | donor | 36,63 | 6,16 | -0,25 | 2,91 | 0,735863 | NS |
|  |  |  |  |  |  |  |  |
| FHL2_C4 | recipient | 55,19 | 5,34 |  |  |  |  |
|  | donor | 54,69 | 6,01 | 0,50 | 3,12 | 0,531157 | NS |
|  |  |  |  |  |  |  |  |
| FHL2_C5 | recipient | 35,13 | 5,61 |  |  |  |  |
|  | donor | 35,00 | 5,73 | 0,13 | 2,66 | 0,853157 | NS |
|  |  |  |  |  |  |  |  |
| FHL2_C6 | recipient | 39,00 | 5,92 |  |  |  |  |
|  | donor | 37,63 | 4,96 | 1,38 | 2,96 | 0,083236 | NS |
|  |  |  |  |  |  |  |  |
| FHL2_C7 | recipient | 22,13 | 3,98 |  |  |  |  |
|  | donor | 21,50 | 3,12 | 0,63 | 2,09 | 0,250984 | NS |
|  |  |  |  |  |  |  |  |
| FHL2_C8 | recipient | 14,38 | 2,75 |  |  |  |  |
|  | donor | 13,88 | 2,03 | 0,50 | 1,63 | 0,239558 | NS |
|  |  |  |  |  |  |  |  |
| FHL2_C9 | recipient | 38,19 | 8,89 |  |  |  |  |
|  | donor | 33,94 | 4,81 | 4,25 | 9,31 | 0,087917 | NS |
|  |  |  |  |  |  |  |  |
| FHL2_C10 | recipient | 11,56 | 3,48 |  |  |  |  |
|  | donor | 10,13 | 1,59 | 1,44 | 3,39 | 0,110082 | NS |
|  |  |  |  |  |  |  |  |
| TRIM_C1 | recipient | 17,38 | 4,33 |  |  |  |  |
|  | donor | 16,50 | 3,12 | 0,88 | 1,96 | 0,094701 | NS |
|  |  |  |  |  |  |  |  |
| TRIM_C2 | recipient | 11,25 | 2,96 |  |  |  |  |
|  | donor | 10,75 | 2,14 | 0,50 | 2,07 | 0,348275 | NS |
|  |  |  |  |  |  |  |  |
| TRIM_C3 | recipient | 20,19 | 6,01 |  |  |  |  |
|  | donor | 20,63 | 5,74 | -0,44 | 3,35 | 0,608607 | NS |
|  |  |  |  |  |  |  |  |
| TRIM_C4 | recipient | 35,25 | 7,44 |  |  |  |  |
|  | donor | 35,56 | 5,70 | -0,31 | 3,84 | 0,749422 | NS |
|  |  |  |  |  |  |  |  |
| TRIM_C5 | recipient | 34,63 | 7,51 |  |  |  |  |
|  | donor | 35,94 | 6,77 | -1,31 | 3,09 | 0,110198 | NS |
|  |  |  |  |  |  |  |  |
| TRIM_C6 | recipient | 29,81 | 8,08 |  |  |  |  |
|  | donor | 30,06 | 6,60 | -0,25 | 3,91 | 0,801478 | NS |
|  |  |  |  |  |  |  |  |
| **TRIM_C7** | recipient | **31,81** | **8,95** |  |  |  |  |
|  | donor | **32,38** | **7,45** | **-0,56** | **4,34** | **0,611346** | NS |
|  |  |  |  |  |  |  |  |
| TRIM_C8 | recipient | 22,44 | 6,78 |  |  |  |  |
|  | donor | 22,13 | 5,49 | 0,31 | 3,30 | 0,710227 | NS |
|  |  |  |  |  |  |  |  |
| **KLF_C1** | recipient | **5,94** | **3,17** |  |  |  |  |
|  | donor | **5,56** | **3,03** | **0,38** | **1,67** | **0,382805** | NS |
|  |  |  |  |  |  |  |  |
| KLF_C2 | recipient | 2,31 | 1,96 |  |  |  |  |
|  | donor | 2,75 | 2,08 | -0,44 | 3,18 | 0,590510 | NS |
|  |  |  |  |  |  |  |  |
| KLF_C3 | recipient | 5,38 | 2,78 |  |  |  |  |
|  | donor | 5,25 | 2,57 | 0,13 | 1,78 | 0,783122 | NS |
|  |  |  |  |  |  |  |  |
| KLF_C4 | recipient | 2,19 | 1,52 |  |  |  |  |
|  | donor | 1,81 | 1,64 | 0,38 | 1,31 | 0,270211 | NS |
|  |  |  |  |  |  |  |  |
| **C1orf_C1** | recipient | **81,31** | **6,86** |  |  |  |  |
|  | donor | **73,13** | **11,44** | **8,19** | **6,91** | **0,000264** | **0,008441** |
|  |  |  |  |  |  |  |  |
| C1orf_C2 | recipient | 72,06 | 9,36 |  |  |  |  |
|  | donor | 58,63 | 11,86 | 13,44 | 7,54 | **0,000003** | **0,00011** |
|  |  |  |  |  |  |  |  |
| C1orf_C3 | recipient | 74,94 | 9,21 |  |  |  |  |
|  | donor | 61,19 | 12,61 | 13,75 | 7,78 | **0,000004** | **0,000121** |
|  |  |  |  |  |  |  |  |
| **Bold** -CpGs included in the original prediction model | | | | | |  |  |
| **Red** - statistically significant differences | | | | |  |  |  |
| NS not significant | |  |  |  |  |  |  |
